# Restitution of Cervical Lordosis Following Anterior Cervical Discectomy and Fusion Using a Fixed Lordotic Angle Cage

**DOI:** 10.7759/cureus.78278

**Published:** 2025-01-31

**Authors:** Goran Lakicevic, Sandra Lakicevic, Bruno Splavski

**Affiliations:** 1 Department of Neurosurgery, University Clinical Hospital Mostar, Mostar, BIH; 2 Department of Neurology, University Clinical Hospital Mostar, Mostar, BIH; 3 Department of Neurological Surgery, Dubrovnik General Hospital, Dubrovnik, HRV; 4 Department of Surgery, School of Medicine, University of Mostar, Mostar, BIH

**Keywords:** cervical lordosis, cobb angle, fixed-angle lordotic cage, harrison back angle, spinal curvature radiographic measuring

## Abstract

Objectives: Degenerative spine disease can result in loss of cervical lordosis. It may lead to spine misalignment, which can be evaluated using quantitative measurements of the Cobb and Harrison back (posterior tangent angle, PTA) angles. Our objective was to investigate the relationship between cervical curvature and the application of wedge-shaped allografts with a predetermined inclination angle in anterior cervical discectomy and fusion (ACDF) surgery in patients with cervical degenerative disease. Additionally, we aimed to evaluate the advantages of this technique in restoring cervical lordosis.

Materials and methods: During a two-year study at a single institution, we performed one-level ACDF on 60 patients using a wedge-shaped fixed-angle allograft with a preplanned inclination angle of 7°. We analyzed changes in the preoperative and postoperative Cobb and PTA angles with standard statistics.

Results: Cobb angle values of the entire cervical segment were increased in 75% of patients after the surgery. Half of the patients had PTA values increased after surgery. There was a significant difference in the mean Cobb and PTA values before and after surgery.

Conclusion: Considering the findings of this study, an ACDF using a fixed lordotic angle wedge-shaped carbon allograft consistently restores the physiological alignment of the cervical spine and reestablishes cervical lordosis.

## Introduction

Degenerative disc disease affecting the cervical spine can cause loss of cervical lordosis, instability in the vertebral dynamic segments, and cervical spondylotic myelopathy [1]. Bone-decompression spinal surgery is intended to relieve the spinal canal's neural elements, preserve or restore cervical lordosis, and stabilize the cervical spine [2-4].

Radiological assessment of the functional capacity of the cervical spine is necessary before and after anterior cervical discectomy and fusion (ACDF). It involves lateral X-rays in the neutral position, flexion and extension, and measurement of the Cobb angle as the most widely used method to quantify the magnitude of cervical spine lordosis or kyphosis in the sagittal plane [5-8]. Cobb’s method includes determining the curves of the upper and lower vertebral bodies (i.e., the bodies with the most tilted endplates) with the maximum inclination toward the convexity of the curve. Hence, the degree of lordosis or kyphosis is determined by the angle between the C2 and C7 vertebral bodies [5].

Measuring the curvature of the cervical spine can also be done using the Harrison back angle method (the posterior tangent angle, PTA), which involves determining the posterior tangents of each vertebral body drawn at the posterior aspects of C2 and C7, with lines that go through the bodies’ posterior edge [9]. The angle of curvature is then calculated as the angle between these tangents, with positive values indicating lordosis and negative ones indicating kyphosis [7,9,10]. The calculation of the Harrison back angle has a lower standard error when compared to the Cobb method and more accurately describes cervical spine segmental curvature [9].

The aim of this study was threefold: 1) to compare two different methods of measuring the preoperative and postoperative radiographic statuses of the cervical vertebral dynamic segments assessed by lateral radiography obtained in a neutral position; 2) to explore the potential correlation between the angles of cervical curvature and the inclination angle of the fixed lordotic angle wedge-shaped allograft frequently employed in ACDF surgery; and 3) to identify possible benefits of using such an allograft in the postoperative restoration of cervical lordosis.

## Materials and methods

During the two years (January 2017 to December 2019), a single-institution observational prospective cohort study was performed on a consecutive series of patients who underwent a one-level ACDF using a wedge-shaped carbon allograft with a fixed angle of inclination of 7°. The standard and large-size variants of the allograft ranged from 4 to 8 mm in anterior height, depending on the patient's cervical spine anatomy.

Adult patients of both genders consecutively operated on for a degenerative spinal disease on a single segment of the cervical spine within two years were included in the study. The patients excluded from the study were those who had undergone surgery on multiple levels, as well as those who had experienced traumatic injury and had metastatic disease or other nondegenerative disease of the cervical spine. The neuroradiological protocol included standard lateral X-rays taken before, six months, and two years (24 months) after ACDF surgery.

If necessary, computerized tomography scans and cervical spine magnetic resonance imaging were taken before and after the surgery. The cervical spine configuration was defined as lordosis, straight spine, or kyphosis. Positive values within the range of standard angle values indicated physiological lordosis, while negative values indicated kyphosis. Preoperative and postoperative cervical spine configuration quantitative analysis was achieved by measuring the overall and segmental angles of the cervical spine of each patient (Cobb angle and Harrison back angle: the PTA of the vertebral body) using a digitally processed X-ray program that can calculate angles based on standard preoperative and postoperative lateral X-rays of the cervical spine. The average angles were identified, as were the range of standard angle values to which each patient belonged. The extent of postoperative change in the curvature of the cervical spine was analyzed by comparing the measured angles to average values and to the range of standard angle values. The risk of bias in interpreting the results was eluded by two independent reviewers who were engaged in angle measuring.

The surgical outcome was assessed at 24 months using Odom criteria, which were classified into four groups: excellent, good, partially successful, and unsuccessful [11]. The investigated sample was intended to be homogeneous in terms of demographics and radiological features. The patients were radiologically followed up for two consecutive years after surgery to assess the changes in cervical configuration and the degree of osseous fusion. The Hospital Research and Ethics Committee approved the study prospectively (approval no. 5474 Klinicka Bolnica Mostar).

Statistical analysis

Parametric and nonparametric tests for dependent samples were used. A t-test for the dependent sample was performed to analyze the changes in the Cobb and Harrison back angles before and after surgery concerning the fixed angle of allograft inclination since it was repeatedly measured in the same group of patients. The strength, direction, and significance of the correlation of the observed variables were analyzed using the Pearson correlation coefficient. The level of statistical significance was set at p = 0.05, and p-values that could not be expressed up to three decimals were expressed as p < 0.001.

Statistical Package for the Social Sciences for Windows (version 17.0, SPSS Inc. Chicago, IL) and Microsoft Excel (version of Office 2007, Microsoft Corporation, Redmond, WA) were used in the statistical analysis of the obtained data.

## Results

**Figure 1:**
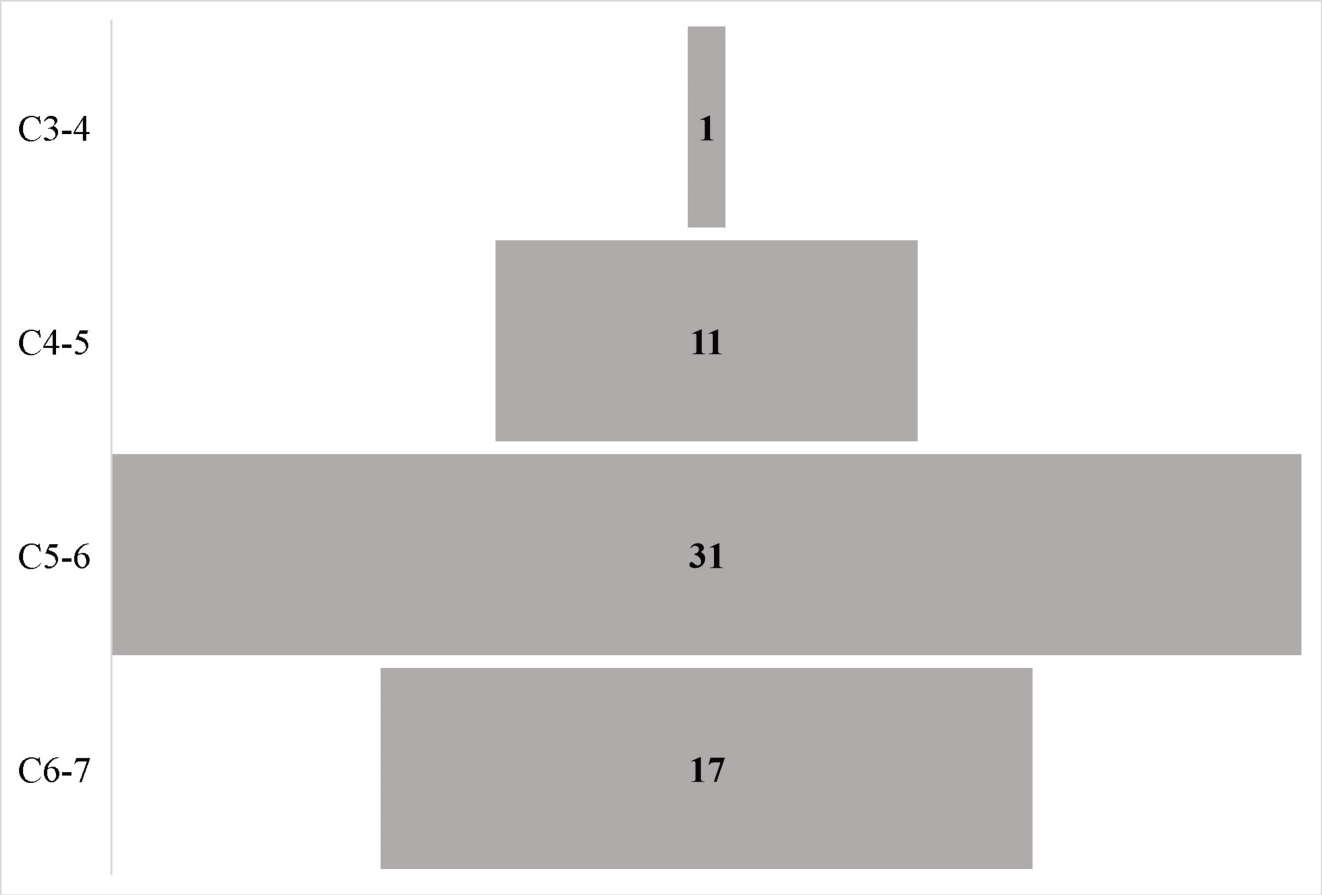
The distribution of vertebral segments operated on

Sixty patients underwent ACDF surgery at different cervical levels and were recruited. There was a 1:1 female-to-male ratio. The average age was 49.1 ± 9.7 years. The majority (31/60) were operated at the C5/C6 level, followed by the C6/C7 level (17 patients), the C4/C5 level (11/60), and the C3/C4 level (1/60). The distribution of vertebral segments operated on is depicted in Figure 1.

Comparison of changes in the Cobb angle before and after surgery

In this series, a Cobb angle range of 26.8° ± 9.72° is considered the range of standard angle values, representing the ordinary configuration of the entire spine. The mean Cobb angle for the entire cervical spine was 17.08° before, 36.52° at six months, and 35,92° at 24 months after surgery. Out of the 60 patients evaluated, 29 (48.3%) had Cobb angle within the range of standard values before surgery, while 31 (51.7%) had values outside the standard range. After the surgery, this number increased to 45 (75.0%) patients, while those who had angle values outside the standard range reduced to 15 (25.0%). A statistically significant difference was found between patients with mean angle values within and outside the standard range of angle values before and after surgery (p = 0.003). Table 1 shows the distribution of patients based on their mean Cobb angle and the range of standard angle values they belong to before and after surgery at six and 24 months.

**Table 1:** The distribution of patients according to the mean Cobb angle values and the range of standard angle values

Range of standard angle values (26.8° ± 9.72°)	Mean angle value, f (%)		
	Before surgery (17.08°)	Six months after surgery (36.52°)	24 months after surgery (35.92°)
Outside the range	31 (51.7)	15 (25.0)	15 (25.0)
Within the range	29 (48.3)	45 (75.0)	45 (75.0)

The mean preoperative Cobb angle between the C2 and C7 body for the entire cervical spine was 19.9° ± 10.7° and 26° ± 8.8° at six months postoperatively, which was a statistically significant difference (t = -6.420; p < 0.001). The mean preoperative Cobb angle was 3.98° for each cervical segment, and this increased from 3.02° to 7° six months after surgery, which was also a statistically significant difference (p = 0.002). The minimum increase was 0.6°, and the maximum was 21.3°, with a segmental Cobb angle increase of 8.6 ± 6.1° at the C5/C6 level (Table 2).

**Table 2:** The changes of mean Cobb angle at six months after surgery according to the level operated on ^*^p = 0.002

Angle change	Number of patients (%)	Operated level			F^*^	p
		C4/C5	C5/C6	C6/C7		
Increase	51 (75%)	7.1° ± 4.0°	8.6° ± 6.1°	7.8° ± 4.5°	0.319	0.729
Decrease	9 (15%)	8.2° ± 0.9°	5.6° ± 5.0°	6.1° ± 4.7°	0.230	0.801

In particular, a statistically significant postoperative increase in the mean Cobb angle for each cervical segment was found in patients whose surgery was performed at the C5/C6 (p < 0.001) and C6/C7 (p = 0.001) levels, as shown in Table 3.

**Table 3:** The mean Cobb angle before and at six months after surgery depending on the level operated on ^*^p < 0.001

Operated level	Before surgery	After surgery	t^*^	p
C4/C5	18.5° ± 12.1°	22.8° ± 7.5°	-2.012	0.072
C5/C6	20.5° ± 11.2°	26.8° ± 10.1°	-4.461	<0.001
C6/C7	19.8° ± 8.2°	25.9° ± 6.8°	-3.999	0.001

The analysis of changes in Cobb angle showed that in 51/60 (85%) patients, the mean angle increased after surgery, with a statistically significant improvement of cervical lordosis (χ^2^ = 29.400; p < 0.001). In nine patients (15%), a decrease in mean Cobb angle was found after surgery regardless of the level operated on.

Comparison of changes in the Harrison back angle before and after surgery

The range of Harrison back angle (PTA) of 34.5° ± 9.82° is considered the range of standard angle values denominating ordinary configuration of the cervical spine. In this series, the mean angle values of the PTA ranged from 24.68° to 44.32° at six months and 43.2° at 24 months after surgery. Eleven (18.3%) patients had PTA within the range of standard angle values before surgery, while 49/60 (81.7%) patients had angle values outside the range of standard values. More than half (31/60; 51.73%) of the patients had PTA within the range of standard angle values after surgery, while 29 (48.3%) patients had PTA values outside the range.

Table 4 shows that there was a statistically significant difference in the PTA of patients whose mean angle values were within the standard range compared to those whose values were outside the standard range before, six months, and 24 months after surgery (p < 0.001).

**Table 4:** Distribution of patients according to the range of standard angle values of the Harrison back angle (PTA) PTA: posterior tangent angle

Standard PTA value (34.5° ± 9.82°)	Mean angle value, f (%)		
	Before surgery (24.68º)	Six months after surgery (44.32°)	24 months after surgery (43.62°)
Within the range	11 (18.3)	31 (51.7)	31 (51.7)
Outside the range	49 (81.7)	29 (48.3)	29 (48.3)

The mean preoperative PTA between the C2 and C7 was 17.4°, increasing to 28.4° for the entire cervical spine at six months after surgery. A statistically significant difference in average PTA was identified before and after surgery (t = -7.707; p < 0.001).

The mean preoperative PTA value was 3.48° for each cervical segment, which increased by 3.52° after surgery. There was a medium-strong, positive, and statistically significant correlation between the mean segmental PTA before and after surgery. In particular, a statistically significant postoperative increase in the mean segmental PTA was found in patients whose surgery was performed at the C5/C6 (p < 0.001) and C6/C7 levels (p < 0.001), as shown in Table 5.

**Table 5:** The mean segmental Harrison back angle before and at six months after surgery according to the level operated on ^*^p < 0.001

Operated level	Before surgery	After surgery	t*	p
C4/C5	23.0° ± 16.4°	28.2° ± 10.8°	-1.924	0.083
C5/C6	15.2° ± 11.7°	27.6° ± 11.7°	-5.703	<0.001
C6/C7	18.5° ± 14.5°	30.0° ± 12.3°	-5.009	<0.001

Surgical outcome according to Odom criteria

Out of the total number of patients, surgical outcome was assessed as excellent in 26 (43.3%) cases, good in 25 (41%) cases, partially successful in seven (11.7%) cases, and unsuccessful in two (3.3%) cases. The majority of patients (50 out of 60 patients; 85%) had excellent or good surgical outcomes. There was no significant difference in surgical outcomes based on the cervical level operated on (p = 0.876).

## Discussion

This study assessed the effectiveness of a wedge-shaped fixed-angle lordotic cage in preserving postoperative cervical lordosis based on radiological and clinical outcomes in patients with cervical degenerative disease.

Understanding spinal kinematics is essential for diagnosing and treating degenerative spinal conditions [12]. Indeed, cervical lordotic alignment is important in maintaining the sagittal balance of the entire spine [13], which is decisive in comprehending the biomechanics of spinal pathologies, particularly in aging individuals [14]. However, while achieving spine stability in the sagittal plane (sagittal balance) has become a recognized and important issue in spinal surgery [14], there is no generally accepted standard algorithm for measuring cervical spine curvature in the sagittal plane and assessing spinal stability. Routine lateral radiographs are commonly used to evaluate cervical alignment, utilizing the Cobb and Harrison back angle (PTA) to measure cervical lordosis [15].

ACDF is a regularly used surgical procedure to treat degenerative cervical spine disease [16,17]. It provides sufficient neural decompression and restores cervical lordosis to its optimal configuration, resulting in solid bone fusion and spinal stability [18]. Hu et al. [19] found that correcting the alignment of the cervical spine can lead to better results for patients undergoing ACDF surgery for one or two levels. Schulz et al. [20] found that allografts are safe and effective in improving clinical and radiologic outcomes. Kim et al. [21] confirmed that ACDF was effective in restoring cervical sagittal alignment if no preoperative cervical deformity existed. Therefore, ACDF remains an efficient approach to deal with most cervical spine symptomatology, including degenerative diseases [22].

Comparison of changes in the Cobb angle before and after surgery

We observed a strong, statistically significant positive correlation (p < 0.001) when comparing the mean Cobb angle before and after surgery for the entire cervical segment. This was also true for each specific cervical segment (p < 0.002), confirming that an increase in the lordotic angle of one cervical segment will lead to an increase in the lordosis of the entire cervical spine. Most patients (75%) achieved standard Cobb angle values following surgery, reflecting a restoration of physiological lordosis. Concurrently, the proportion of patients exhibiting angles outside the normal range, indicative of kyphosis, was significantly reduced to 25%. A statistically significant difference was observed between these patient groups (p = 0.003).

When performing ACDF in our series, we utilized a wedge-shaped carbon allograft set at a predetermined and fixed inclination angle of 7° in lordosis. We have opted for a 7° lordotic wedge-shaped cage instead of a standard cage during the ACDF to assess its potential benefits on the postoperative restoration of cervical lordosis in patients with spondylosis, an outcome that is typically well documented when a standard cage is used for disc herniation.

However, in a small percentage of patients (15%) in our series, we observed a decrease in the mean postoperative Cobb angle, where there was no restoration of lordosis. We believe this is probably triggered by cage subsidence, which decreases the Cobb angle, increases the stress on the anterior fixation system, and may cause biomechanical cervical instability [23,24]. The results obtained here were in line with those reported in the relevant literature, confirming that most patients experienced a significant increase in the mean Cobb angle after ACDF surgery [25-27].

Comparison of changes in the Harrison back angle before and after surgery

According to Harrison et al., the PTA method is the best way to calculate the curvature of the cervical spine. It is more accurate for measuring the changes in the sagittal profile for each cervical segment than Cobb's method, which provides a better global curvature measuring of angles between the C2 and C7 bodies of the cervical spine sagittal profile [9].

In our series, the number of patients whose PTA values were within the range of standard angle values substantially increased after the surgery, which was statistically significant (p < 0.001). A statistically significant increase was found between the mean PTA before and after surgery in the great majority of patients (88.3%). A medium-strong, positive, and statistically significant correlation existed between mean PTA before and after surgery (p < 0.001). The highest mean increase was found in patients who were operated on the C5/C6 level, and the lowest was found in those who were operated on the C4/C5 level (Table 5). These findings align with those documented in the literature supporting lordotic allografts, resulting in a mean increase of the PTA and restitution of cervical lordosis [16,27]. However, preoperative kyphosis can also be corrected with a standard cage [28]. Nonetheless, cage geometry has a noteworthy impact on the spinal alignment after instrumented fusion, where wedge-shaped lordotic/hyperlordotic cages should be preferred for restoring spinal sagittal alignment [29,30].

Comparison of the Cobb and Harrison back angle changes before and after surgery depending on the allograft inclination angle degree

When performing ACDF in our series, we utilized a wedge-shaped carbon allograft set at a predetermined and fixed inclination angle of 7° in lordosis. We have opted for a 7° lordotic wedge-shaped cage instead of a standard cage during the ACDF to assess its potential benefits on the postoperative restoration of cervical lordosis in patients with spondylosis, an outcome that is typically well documented when a standard cage is used for disc herniation.

To gain a better insight into the overall and segmental curvature of the cervical spine after surgery, changes in the Cobb and Harrison back angles were analyzed before, six months, and 24 months after surgery to the constant inclination angle of a cage. Using this implant, we increased the mean segmental Cobb angle by 3.02° at six months, which was a statistically significant increase (p = 0.002). There was also a strong, positive, and statistically significant correlation between the mean Cobb angle before and six months after surgery for the entire cervical spine (p < 0.001). In our series, a significant postoperative increase in the mean segmental Harrison back angle was also observed. Therefore, ACDF with a wedge-shaped allograft having a fixed inclination angle has a positive effect on preserving the Cobb angle and PTA within the range of standard angle values. These measures are expressed in degrees, providing numeric values rather than descriptive ones.

At up to 24 months, both angle values showed a significant improvement compared to baseline values. There was no radiological evidence of instability and no statistically significant difference in outcomes at any time after surgery. Therefore, using such an allograft is beneficial in the postoperative restoration of cervical lordosis.

Surgical outcome according to Odom criteria

To further assess the safety and effectiveness of the procedure after 24 months, we conducted a clinical patient evaluation using Odom criteria [11]. The results showed that the great majority of patients had excellent or good surgical outcomes after ACDF with a wedge-shaped carbon allograft.

Study limitations

Given its single-institution design, a relatively limited number of patients, and a short follow-up period of two years, this study has noteworthy limitations that should be acknowledged. The major limitation is the absence of a control group of patients with a standard ACDF cage for comparison.

## Conclusions

Considering the findings of this study, an ACDF using a fixed lordotic angle wedge-shaped carbon allograft consistently restores the physiological alignment of the cervical spine and reestablishes cervical lordosis.
